# Transient increase in atherosclerotic plaque macrophage content following *Streptococcus pneumoniae* pneumonia in ApoE-deficient mice

**DOI:** 10.3389/fcimb.2023.1090550

**Published:** 2023-03-23

**Authors:** Rohit Bazaz, Helen M. Marriott, Carl Wright, Janet Chamberlain, Laura E. West, Catherine Gelsthorpe, Paul R. Heath, Afsaneh Maleki-Dizaji, Sheila E. Francis, David H. Dockrell

**Affiliations:** ^1^ Division of Infection, Immunity and Respiratory Medicine, Faculty of Biology, Medicine and Health, University of Manchester, Manchester Academic Health Science Centre, Manchester, United Kingdom; ^2^ Department of Infectious Diseases, Wythenshawe Hospital, Manchester University NHS Foundation Trust, Manchester, United Kingdom; ^3^ Department of Infection, Immunity and Cardiovascular Disease, University of Sheffield, Sheffield, United Kingdom; ^4^ Sheffield Institute for Translational Neuroscience, University of Sheffield, Sheffield, United Kingdom; ^5^ AF ChemPharm Ltd, Sheffield, United Kingdom; ^6^ MRC Centre for Inflammation Research, University of Edinburgh, Edinburgh, United Kingdom

**Keywords:** pneumococcus, myocardial infarction, atherosclerosis, acute coronary syndrome, statin

## Abstract

**Introduction:**

Despite epidemiological associations between community acquired pneumonia (CAP) and myocardial infarction, mechanisms that modify cardiovascular disease during CAP are not well defined. In particular, largely due to a lack of relevant experimental models, the effect of pneumonia on atherosclerotic plaques is unclear. We describe the development of a murine model of the commonest cause of CAP, *Streptococcus pneumoniae* pneumonia, on a background of established atherosclerosis. We go on to use our model to investigate the effects of pneumococcal pneumonia on atherosclerosis.

**Methods:**

C57BL/6J and ApoE^-/-^ mice were fed a high fat diet to promote atherosclerotic plaque formation. Mice were then infected with a range of *S. pneumoniae* serotypes (1, 4 or 14) with the aim of establishing a model to study atherosclerotic plaque evolution after pneumonia and bacteremia. Laser capture microdissection of plaque macrophages enabled transcriptomic analysis.

**Results:**

Intratracheal instillation of *S. pneumoniae* in mice fed a cholate containing diet resulted in low survival rates following infection, suggestive of increased susceptibility to severe infection. Optimization steps resulted in a final model of male ApoE^-/-^ mice fed a Western diet then infected by intranasal instillation of serotype 4 (TIGR4) *S. pneumoniae* followed by antibiotic administration. This protocol resulted in high rates of bacteremia (88.9%) and survival (88.5%). Pneumonia resulted in increased aortic sinus plaque macrophage content 2 weeks post pneumonia but not at 8 weeks, and no difference in plaque burden or other plaque vulnerability markers were found at either time point. Microarray and qPCR analysis of plaque macrophages identified downregulation of two E3 ubiquitin ligases, Huwe1 and Itch, following pneumonia. Treatment with atorvastatin failed to alter plaque macrophage content or other plaque features.

**Discussion:**

Without antibiotics, ApoE^-/-^ mice fed a high fat diet were highly susceptible to mortality following *S. pneumoniae* infection. The major infection associated change in plaque morphology was an early increase in plaque macrophages. Our results also hint at a role for the ubiquitin proteasome system in the response to pneumococcal infection in the plaque microenvironment.

## 1 Introduction

Premature mortality due to cardiovascular disease represents a major challenge to sustainable human development. In 2015, there were approximately 18 million deaths globally due to cardiovascular disease and the burden is continuing to increase in low income countries (Clark, 2013; Roth et al., 2017). Low income countries also bear a disproportionate burden of lower respiratory tract infection (Global Burden of Disease Study 2016). Despite the significant burden of these diseases globally and increasing recognition of an association between them, there is a relative lack of understanding of the underlying mechanisms.

Evidence from observational clinical studies suggests that community acquired pneumonia (CAP) is associated with an increased risk of major adverse cardiac events (MACE), including myocardial infarction (MI) (Warren-Gash et al., 2018; Musher et al., 2019). *Streptococcus pneumoniae* is the most commonly identified pathogen causing CAP worldwide (Welte et al., 2012; Said et al., 2013). Pneumococcal pneumonia and sepsis are independent risk factors for the development of MACE following pneumonia (Viasus et al., 2013). Myocardial infarction has been reported to occur in 5-7% of patients hospitalized for pneumococcal pneumonia (Musher et al., 2000; Musher et al., 2007), with the highest risk occurring during the first few days following infection.

Despite the growing body of clinical data that suggests bacterial pneumonia can trigger MI, there is a relative paucity of published experimental data investigating the mechanistic link between these comorbidities. The vast majority of MI episodes occur as a result of thrombotic complications at the site of coronary atherosclerotic plaque rupture (Ambrose et al., 1988; Falk et al., 1995). It has been proposed that the systemic inflammatory response to pneumonia may result in localized inflammatory changes within coronary atherosclerotic plaques, thereby increasing the risk of plaque rupture (Musher et al., 2019). One impediment to developing a more nuanced understanding of these mechanisms is the absence of an appropriate murine model in which to test hypotheses. An animal model of bacterial pneumonia on a background of established atherosclerosis is therefore required to explore if plaque perturbation in the aftermath of pneumonia is the primary mechanism underlying this relationship.

In this study we developed a model of invasive pneumococcal disease on a background of established atherosclerosis and used this to investigate the effects of pneumococcal infection on atherosclerotic plaque development, focusing on markers of plaque vulnerability to rupture. We found that infection resulted in an early but transient increase in plaque macrophage content together with downregulation of two E3 ubiquitin ligases by plaque macrophages although no long term effects on plaques were identified. Atorvastatin initiated following onset of pneumonia had no discernible protective effect on plaque composition.

## 2 Materials and methods

### 2.1 Bacteria

Serotype 1 *S. pneumoniae* (WHO reference laboratory strain SSISP, Statens Institute, Denmark), serotype 4 (TIGR4, American Type Culture Collection CA, USA) or serotype 14 (NCTC11902, Public Health England, UK) were grown as previously described (Dockrell et al., 2003). Prior to their use *in vivo*, all bacterial strains were passaged through C57BL/6J mice (Harlan, UK) by intraperitoneal (i.p.) injection and recovery of bacteria from blood taken at 16 hours post infection and incubated on Columbia blood agar (Canvin et al., 1995).

### 2.2 Mice

All mice were used in compliance with the UK Animals (Scientific Procedures) Act 1986 and Directive 2010/63/EU and all experiments were approved by the animal project review committee of the University of Sheffield and conducted under a Home Office approved animal project license. C57BL/6J mice and ApoE^-/-^ mice (on a C57BL/6J background) were bred in house. All mice were housed in a controlled environment at 22°C with a 12 h light/dark cycle. Prior to and after periods of high fat diet feeding, mice were fed standard chow diet. Mice which were fed a high fat diet were given either 5g/mouse/day of Western diet (20% casein, 16% fat (15% cacao butter, 1% corn oil), 0.25% cholesterol; Abdiets, The Netherlands) or 5g/mouse/day of Paigen diet (18.5% fat, 0.9% cholesterol and 0.5% cholate; Special Diet Services, UK) for 8 weeks from 11-12 wks of age, and then put back on to chow diet. All experiments were conducted at the same time of day to minimize the impact of circadian rhythm and mice were co-housed to minimize microbiome associated variation.

### 2.3 Infection model

Intratracheal instillation of pneumococci, or mock infection with phosphate-buffered saline (PBS), was performed as previously described (Dockrell et al., 2003). Prior to intratracheal instillation, each mouse was anaesthetised using ketamine (100 mg/kg i.p.; Willows Francis, UK) and acepromazine (5mg/kg i.p; C-Vet Veterinary Products, UK). Once the pedal reflex was lost, the neck of each anaesthetised mouse was shaved and a midline incision of approximately 1cm was made on the ventral aspect. The trachea was exposed by dissection of the adjacent tissues with blunt forceps. A 24-gauge catheter (Smiths Medical, OH, USA) was then inserted into the lumen of the trachea, and 20 µl solutions containing either pneumococci or PBS alone were instilled into the lungs using a pipette. Following instillation, the catheter was removed from the trachea and the surrounding tissue pinched together to close the incision. The mice were then left on their backs in an incubator at 33°C and observed closely for any signs of distress until they had fully regained consciousness. Mice undergoing intranasal instillation of pneumococci were anaesthetised using inhaled 4% isoflurane (Zoetis, UK) delivered in 2 l/min O_2_ inside a 1 litre induction chamber until an areflexic state was reached. Instillation was then performed by placing the animal in a supine position and pipetting 50 µl of a solution of pneumococci (or mock infection with 50 µl PBS) onto the nostrils, allowing the mouse to inhale each droplet before introducing the next one. Following either intranasal or intratracheal instillation of bacteria or PBS, mice were continuously observed until full consciousness was regained. They were then inspected every 12 hours during the first 48 hours following instillation and daily thereafter. Any mouse which displayed signs of severe illness following instillation of bacteria was culled by humane methods. Severe illness was defined as any of the following features: severely reduced respiratory rate (0.5s between breaths), severely reduced mobility (mouse only moved when provoked and then only a few steps) or hunched posture. Blood, bronchoalveolar lavage (BAL) fluid and lungs were collected and total and differential cell counts in BAL fluid and viable bacterial count in blood and lung homogenates were measured as previously described (Miles et al., 1938; Dockrell et al., 2003). During experiments assessing atherosclerosis outcomes, only mice surviving to pre specified time points were included in the analyses.

### 2.4 Administration of amoxicillin

A veterinary preparation of amoxicillin was used (Amoxypen 150 mg/ml suspension for injection, Intervet, UK). Both infected and mock infected mice received amoxicillin. The mice were given 3 doses of 100mg/kg amoxicillin s.c. at 12 hourly intervals. The first amoxicillin dose was administered 24 hours post infection or mock infection, immediately after the tail vein snip procedure, if performed.

### 2.5 Atorvastatin administration

Mice receiving atorvastatin were administered powdered Atorvastatin Calcium (Pfizer) in 0.5% methylcellulose by daily gavage.

### 2.6 Analysis of atherosclerosis

Mice were killed by an overdose of i.p. sodium pentobarbital. Blood was collected by cardiac puncture and mice were then perfusion fixed with 10% v/v of formalin buffered saline. The aortic sinus and brachiocephalic artery were dissected, embedded in paraffin and serially sectioned (5 µm). Aortic sinus sections were stained with Miller’s Elastin/Modified Van Gieson (MVG), to enable analysis of atheromatous lesions including lesion area, or Picrosirius Red to measure plaque collagen content. Aortic sinus sections were immunostained for macrophage marker MAC-3, α-smooth muscle actin or cellular proliferation marker Ki67. A detailed description of the immunostaining methods used can be found in the supplementary file (**Supplementary Table 1**). Images of stained histological sections were analysed using NIS-Elements Image Analysis Software (Nikon Instruments, UK). MVG stained aortic sinus sections were used to measure atherosclerotic lesion area which was expressed as a percentage of vessel cross-sectional area (CSA). Four consecutive sections from each aortic sinus were analysed to generate a mean atherosclerotic lesion area/CSA measurement for each mouse. MVG stained sections were also used to measure necrotic core area, as defined by acellular areas within atherosclerotic lesions, as a proportion of total lesion area. Collagen content of atherosclerotic lesions was expressed as area of positive Picrosirius Red staining as a proportion of total lesion area. Similarly, area of positive immunostaining for MAC-3 and smooth muscle actin were expressed as a proportion of total lesion area, while Ki67 immunostaining was expressed as the proportion of atherosclerotic lesion cells staining positive.

### 2.7 Immunofluorescence staining

Aortic sinus sections were stained with anti-serotype 4 pneumococcal antiserum using immunofluorescence to detect bacterial invasion into tissue. Following incubation with the pneumococcal antiserum, sections were stained with Alexa Fluor 568 anti-rabbit IgG secondary antibody and analysed using a wide field fluorescence microscope. A detailed immunofluorescence staining protocol can be found in the Supplementary file.

### 2.8 Laser capture microdissection and RNA extraction

Mice were killed by an overdose of i.p. sodium pentobarbital. The aortic sinus was dissected, embedded in optimal cutting temperature compound (OCT) (Thermo Fisher Scientific, UK) and frozen. Serial sections (7µm) through the aortic sinus were prepared using a cryostat (Leica CM3050 S, Leica, Germany). Every 5^th^ frozen aortic sinus slide was immunostained for the macrophage marker MAC-3 as a ‘guide slide’ to direct laser capture microdissection (LCM) of macrophages on the remaining cryo-sections (Feig and Fisher, 2013). A detailed protocol for MAC-3 immunostaining of cryo-sections can be found in the supplementary file. All aortic sinus cryo-sections which were not immunostained for MAC-3 underwent LCM and were stained with 0.1% w/v Toluidine blue. Sections were fixed in 70% ethanol for 30s, rinsed in DEPC water before staining for 30s in Toluidine blue and rinsing again in DEPC water. Tissue sections were then dehydrated through increasing concentrations of ethanol for 30 seconds each step (70%, 95%, 2 changes of 100%) and xylene for 5 minutes. Slides were then left to air dry before proceeding immediately to LCM. LCM on aortic sinus cryo-sections was performed using the Pixcell II laser-capture microdissection system (Arcturus Engineering, CA, USA) and cells were collected using Capsure Macro caps (Arcturus Engineering). The Pixcell system was set to the following parameters: 40 mW power and 7.5 µm laser spot size. Approximately 2000 MAC-3 positive cells were collected from each aortic sinus. The PicoPure RNA isolation kit (Thermo Fisher Scientific, UK) was used to recover total RNA from cells isolated during LCM, according to the manufacturer’s instructions.

### 2.9 Microarray and pathway analysis

The Affymetrix GeneChip Mouse Gene 2.0 ST Array (Affymetrix, UK) was used to measure whole-transcriptome gene expression in a single hybridisation. The WT Pico Reagent Kit (Affymetrix) was used according to the manufacturer’s instructions to process RNA samples (500pg of RNA from each aortic sinus) obtained following LCM and convert them to double stranded cDNA to act as a template for *in vitro* transcription (IVT). During the IVT step, complimentary RNA (cRNA) was synthesised and also amplified using T7 RNA polymerase. 20 µg of cRNA from each sample was then used to synthesise fragmented and labelled cDNA ready for array hybridisation. The Affymetrix Gene Chip Mouse Gene 2.0 ST Array cartridge was used according to manufacturer’s instructions. Following hybridisation, each microarray cartridge was washed and stained in the GeneChip Fluidics Station 400 (Affymetrix) according to manufacturer’s instructions. The arrays were stained with light sensitive streptavidin phycoerythrin (SAPE), followed by the addition of a biotinylated anti-streptavidin antibody. Finally, the arrays were scanned in the GeneChip 3000 scanner (Affymetrix, UK).

Microarray data were normalised using the Robust Multichip Average method (Bolstad et al., 2003) and then analysed using the Linear Models for Microarray (LIMMA) package in R. Differentially expressed genes between infected and mock infected groups were identified through unpaired t-tests, using the parameters p<0.05 and gene expression log2 fold change >1. The analysis was conducted twice, firstly with False Discovery Rate (FDR <0.05) adjusted p value and the second time with unadjusted p value. The list of differentially expressed genes was then used for pathway analysis to identify the biological pathways that had been significantly perturbed by pneumococcal infection. Kyoto Encyclopedia of Genes and Genomes (KEGG) pathway analysis was performed using the clusterProfiler in R (Yu et al., 2012), with statistical significance set at p<0.05.

### 2.10 Real time quantitative polymerase chain reaction (qPCR)

Quantitative real-time PCR (qPCR) was used to validate microarray differential gene expression results. Firstly, the qScript cDNA Supermix (Quanta Bio, MA, USA) was used according to the manufacturer’s instructions to synthesise single stranded cDNA from each RNA sample obtained following LCM of plaque macrophages. The PCR reaction was run on a 7900T fast real time PCR system (Applied Biosystems, UK) using the GoTaq Probe qPCR Master Mix (Promega) in accordance with the manufacturer’s instructions. Data were normalised to cyclophilin A or 18S. Fold change was calculated using Delta Delta Ct values. The PCR primers used are listed in **Supplementary Table 2**.

### 2.11 Statistics

Statistical analysis was performed using R for the microarray or for all other analyses in Graphpad Prism version 7.0. Data were expressed as mean +/- standard error of the mean (SEM) unless otherwise stated. Comparison between groups employed unpaired t-test unless otherwise stated in figure legends. Statistical significance was defined as p<0.05.

## 3 Results

### 3.1 Intratracheal infection in mice with severe atherosclerosis

**Figure 1:**
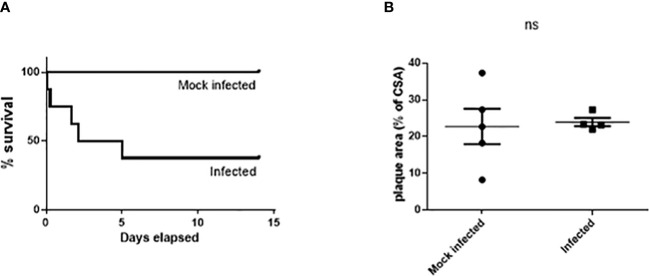
Significant mortality but no significant effect on atherosclerosis burden in male Paigen fed ApoE^-/-^ mice following Type 1 *S. pneumoniae* infection. Cumulative survival **(A)** after intratracheal instillation of 10^7^ cfu serotype 1 *S. pneumoniae* (n=8) or mock infection with PBS (n=5) (p=0.037 logrank test). No significant difference in lesion area in the aortic sinus **(B)**. ns, not significant.

Serotype 1 *S. pneumoniae* was initially used due to its high invasive potential (Sandgren et al., 2004; Sandgren et al., 2005) but preliminary studies showed only 3 out of 8 Paigen diet fed mice (38%) survived to 14 days post infection making this model unsuitable to investigate long term effects of pneumococcal disease. Two mice died within 6 h of infection, 2 were culled 24-72 h post infection and 1 was culled at 5 days due to signs of severe illness (**Figure 1A**). All but 1 of the 6 infected mice which had a blood sample taken at 24 h post infection by tail vein snip were bacteremic, with mean blood viable bacterial count of 5.3 x 10^5^ cfu/ml (range 3.3 x 10^2^ – 1.6 x 10^6^).

There was no significant difference in aortic sinus lesion area between the 2 groups (22.78% ± 4.82 mock infected vs. 23.99% ± 1.15 infected; n=4-5, ns) (**Figure 1B**) in mice which had survived for greater than 72h after infection/mock infection.

### 3.2 Intranasal infection in Western diet fed mice

**Figure 2:**
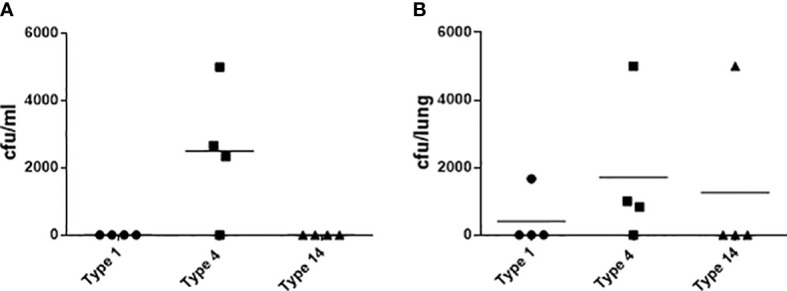
Microbiological outcomes in 10 week old female C57BL/6J mice 24 hours after intranasal instillation of *S. pneumoniae*. Bacteria in blood **(A)** and lung homogenates **(B)** following infection with 10^7^ cfu of type 1, 4 or 14 pneumococci (n=4).

We then adjusted the diet to a Western diet to develop a model with less extensive (sub-maximal) atherosclerotic plaque formation, thus enabling more potential for enhancement by infection, and also with the aim of reducing early infection-related mortality that was potentially attributable to the Paigen diet. In addition to atherosclerosis, high-fat diet fed ApoE^-/-^ mice have been shown to be a model of non-alcoholic steatohepatitis (NASH) with hepatic fibrosis, a condition which is associated with an increased risk of postoperative death following major surgery in humans (Zarzavadjian Le Bian et al., 2012; Schierwagen et al., 2015). Ketamine and acepromazine are metabolized primarily by the liver, and therefore liver dysfunction may have potentiated their effects in this model, possibly contributing to the early post-procedure mortality (Soleimanpour et al., 2015). One of the reasons therefore to switch to the Western diet, which does not contain any cholate and which has a lower cholesterol content than the Paigen diet, was to mitigate the potentially increased susceptibility to anaesthesia. The model was further modified by the replacement of intratracheal with intranasal instillation, thus modifying the general anaesthetic requirements which may also have contributed to the early mortality demonstrated following intratracheal instillation. We next screened pneumococcal strains with relatively high invasive potential to identify one that resulted in high rates of bacteremia following intranasal instillation. Of the serotype 4 infected mice, 3 out of 4 were bacteremic whereas there were no bacteremic mice amongst those infected with serotypes 1 and 14 (**Figure 2A**). Three out of 4 mice infected with serotype 4 *S. pneumoniae* had detectable pneumococci in the lungs compared to 1 out of 4 of those infected with serotypes 1 and 14 (**Figure 2B**).

Having demonstrated relatively high rates of bacteremia following intranasal instillation of serotype 4 *S. pneumoniae*, we then sought to optimize the infecting dose in Western diet fed ApoE^-/-^ mice to maximize the percentage of mice with bacteremia. The percentage of mice with bacteremia following the 10^5^ cfu dose was 100%, compared with 40% (5 x 10^4^ cfu) and 20% (10^4^ cfu) for the other doses (n=3-5) (**Supplementary Figure 1**).

The survival rate of Western diet fed ApoE^-/-^ mice infected with the 10^5^ cfu dose was then assessed. Within 72 h of infection, all mice (n=8) either died or had to be culled (2 died, 6 culled) due to developing signs of severe illness. Two of these mice were not bacteremic at 24 hours post infection. Survival assessments in smaller cohorts (n=3) of Western diet-fed ApoE^-/-^ mice infected with 5 x10^4^ cfu and 10^4^ cfu also demonstrated high mortality rates within 72 hours of infection (3 and 2 were culled respectively).

### 3.3 Resolving model of invasive disease

As doses lower than 10^5^ cfu resulted in relatively low rates of bacteremia, the model was further modified to combine invasive disease with subsequent antimicrobial treatment to enhance survival despite establishment of pneumonia and early bacteremia, thus replicating the clinical situation. The use of antibiotics was also predicted to allow for the instillation of a higher dose of pneumococci, and 5 x10^5^ cfu was chosen with the aim of generating greater blood bacterial counts than those seen with the 10^5^ cfu dose.

**Figure 3:**
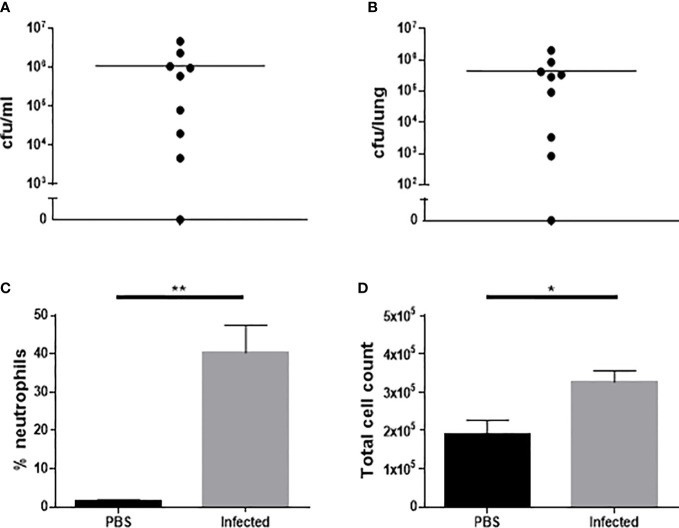
Characterization of microbiological and inflammatory features of Western diet-fed male ApoE^-/-^ mice 24 hours after infection with 5 x10^5^ cfu type 4 *S. pneumoniae* by intranasal instillation. Bacteria in blood **(A)** and lung homogenates **(B)** following infection with *S. pneumoniae* (n=9). Differential BAL fluid cell count expressed as % neutrophils **(C)** following infection or mock infection with PBS (n=8-9, **p<0.005, unpaired t-test with Welch’s correction). Total BAL fluid cell count **(D)** following infection or mock infection with PBS (n=8-9, *p<0.05).

Eight out of 9 mice (88.9%) were bacteremic and eight out of 9 (88.9%) had detectable pneumococci in the lungs at 24 h post infection (**Figures 3A, B**). The single mouse in this cohort which was not bacteremic did have detectable bacteria in the lungs and 9% BAL fluid differential neutrophil count, suggestive of lung infection with associated inflammatory response. Compared to mock infected mice, the infected mice demonstrated a significant increase in total and differential (% neutrophil) BAL fluid cell counts, consistent with the development of pneumonia (**Figures 3C, D**).

The introduction of antimicrobial therapy was the final modification to the model. Infected mice were given 3 x 100mg/kg doses of amoxicillin subcutaneously (s.c.) at 12 hourly intervals with the first dose given at 24 h post infection. Twenty-six mice were infected using this model, three were culled at 36, 48 and 72 h post-infection respectively due to signs of severe illness, with the rest surviving throughout the 2 wk study. The model therefore resulted in a 2 wk post-infection survival rate of 88.5%.

### 3.4 Atherosclerosis development after western diet feeding

Atherosclerotic lesion development was assessed in the aortic sinus of Western diet-fed ApoE^-/-^ mice at the end of the 8 week high fat-diet feeding period (**Supplementary Figure 2**). All 5 mice developed at least one large advanced atherosclerotic plaque at the aortic root with extracellular lipid and areas of fibrous cap formation.

### 3.5 Effect of pneumococcal pneumonia on atherosclerotic plaque burden and composition

The final optimized infection plus atherosclerosis model can be summarized as follows:

1. Male ApoE^-/-^ mice aged 11-12 weeks fed a Western diet for 8 weeks prior to infection.

2. Intranasal instillation of 5x10^5^ cfu serotype 4 (TIGR4) *S. pneumoniae.*

3. Three doses of s.c. 100 mg/kg amoxicillin 12 hourly commenced 24 h post infection.

4. Mice maintained on chow diet for the required observation period post infection.

**Figure 4:**
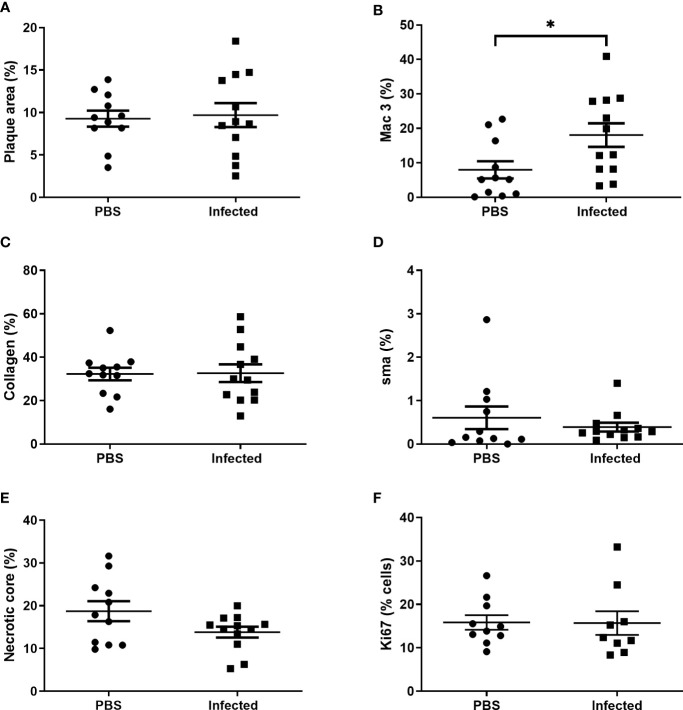
Pneumococcal pneumonia results in increased atherosclerotic plaque macrophage content 2 weeks post pneumonia but no effect on other markers of plaque vulnerability. Male ApoE^-/-^ mice aged 11-12 weeks were fed a Western diet for 8 weeks prior to intranasal instillation of 5x10^5^ cfu serotype 4 (TIGR4) *S. pneumoniae* or mock infection with PBS. Mice received three doses of s.c. 100 mg/kg amoxicillin 12 hourly commenced 24 h after infection/mock infection and were maintained on chow diet post infection. Aortic sinus lesion area **(A)**, expressed as a % of sinus CSA, in control mice and *S. pneumoniae* infected mice. Plaque macrophage **(B)**, collagen **(C)** and smooth muscle actin **(D)** content expressed as % area of positive staining/total lesion area. Plaque necrotic core area expressed as % of total plaque area **(E)**. Plaque cell replication **(F)** expressed as % of plaque cells staining positive for Ki67. Data were analysed using unpaired t test **(A–C, E, F)** or Mann-Whitney U test **(D)** (n=11-12, *p<0.05).

**Figure 5:**
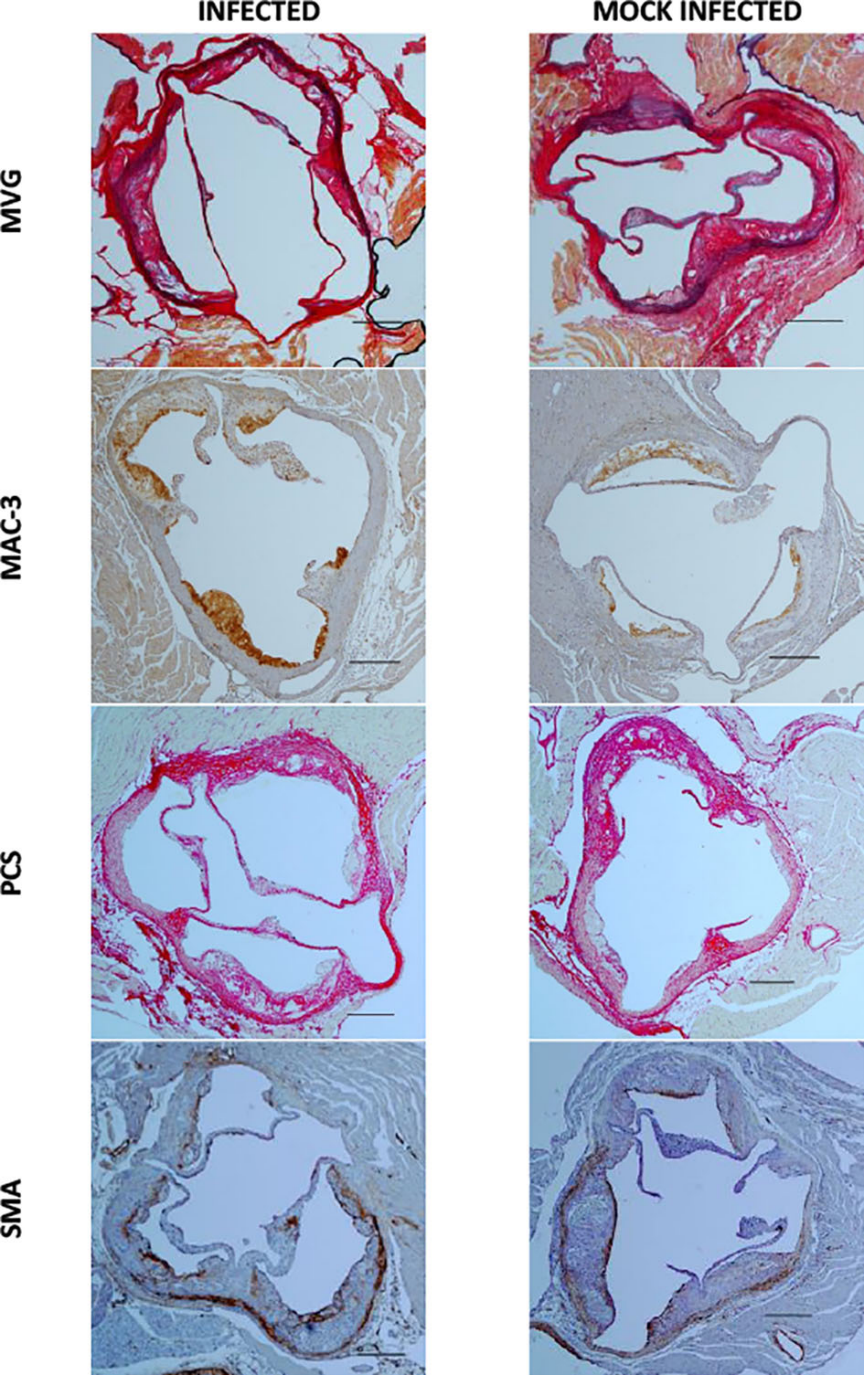
Aortic sinus sections 2 weeks post infection and mock infection. Representative images of MVG, MAC-3, Picrosirius Red (PCS) and smooth muscle actin (SMA) stained aortic sinus sections from *S. pneumoniae* infected (n=12) and PBS mock infected (n=11) ApoE^-/-^ mice, using the optimised model as described in **Figure 4**. Scale bars represent 400 µm.

**Figure 6:**
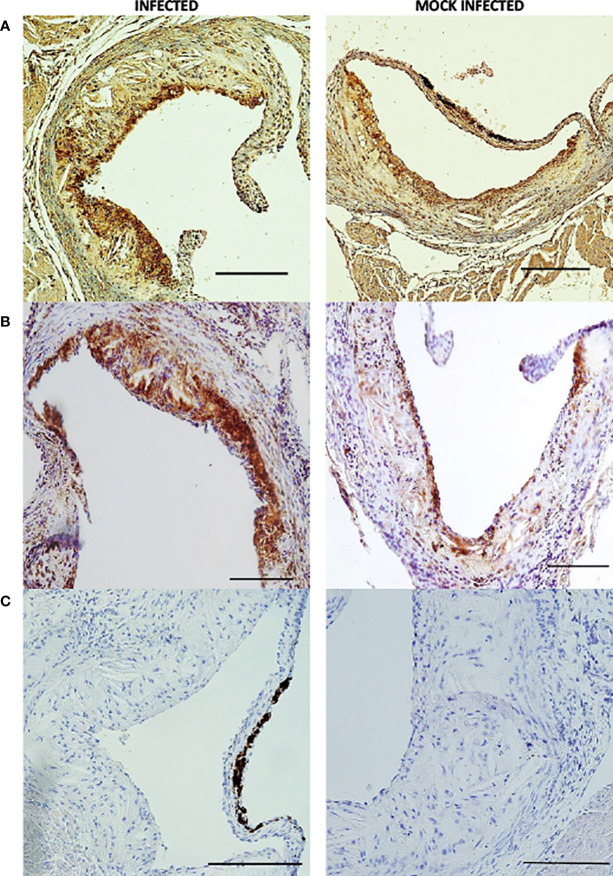
Individual aortic sinus atherosclerotic plaque sections 2 weeks post infection and mock infection. Representative images of MAC 3 **(A, B)** and haematoxylin and eosin **(C)** stained atherosclerotic plaque sections from *S. pneumoniae* infected (n=12) and PBS mock infected (n=11) ApoE^-/-^ mice, using the optimised model as described in **Figure 4**. Scale bars represent 100 µm.

**Figure 7:**
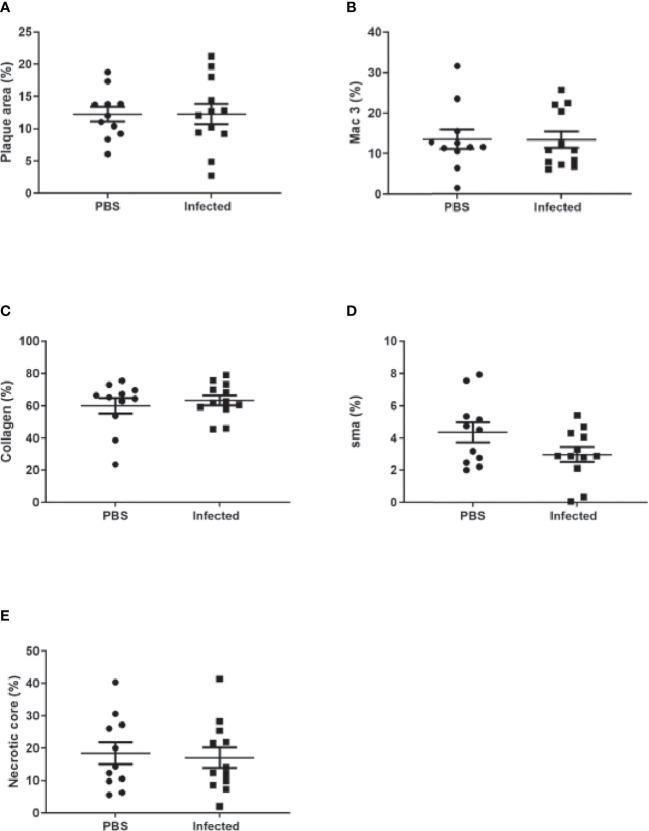
Pneumococcal pneumonia had no effect on aortic sinus plaque size or composition 8 weeks post pneumonia. Quantification of aortic sinus plaque area **(A)**, plaque macrophage **(B)**, collagen **(C)**, smooth muscle **(D)** content and necrotic core area **(E)** in infected and control ApoE^-/-^ mice (n=11-12).

Using this optimized model, we then investigated the effect of pneumococcal pneumonia on aortic sinus atherosclerotic plaque burden and markers of plaque vulnerability to rupture at 2 weeks and 8 weeks post infection. Pneumococcal pneumonia had no effect on aortic sinus plaque area at either time point, but did lead to increased plaque macrophage content 2 weeks post infection (**Figures 4A, B**, **5**, **6**). No difference in plaque macrophage content was observed at the 8 week time point, and no difference in aortic sinus plaque smooth muscle, collagen content or necrotic core size were found at either time point (**Figures 4C–E**, **5**, **7**). Plaque cell proliferation, quantified by the proportion of Ki67-positive plaque cells, was also unaffected by pneumococcal pneumonia at 2 weeks post infection (**Figure 4F**). Brachiocephalic arteries did not develop significant atherosclerotic plaques in our model, nor was there any evidence of inflammatory infiltration in the arterial wall at this site following infection (**Supplementary Figure 3**). Immunofluorescence assays using anti-serotype 4 pneumococcal antiserum showed no positive staining in atherosclerotic plaques or elsewhere in the aortic sinus 2 weeks post infection using our optimized model, and therefore we found no evidence of pneumococcal invasion into atherosclerotic plaques at this time point (**Supplementary Figure 4**).

### 3.6 Transcriptomic analysis of atherosclerotic plaque macrophages following pneumococcal infection

Having demonstrated increased plaque macrophage content 2 weeks post pneumococcal infection, we studied the transcriptional profile of these macrophages. First, to confirm that LCM leads to enrichment of macrophage specific transcripts, RNA was extracted from LCM-derived MAC-3 immuno-positive plaque cells and from whole aortic sinus sections of ApoE^-/-^ mice fed a Western diet for 8 weeks. Using qPCR to compare gene expression, LCM obtained samples demonstrated enrichment of the macrophage specific transcript CD68 and conversely expression of the smooth muscle cell marker ACTA2 was reduced in samples obtained by LCM (**Supplementary Figure 5**).

**Figure 8:**
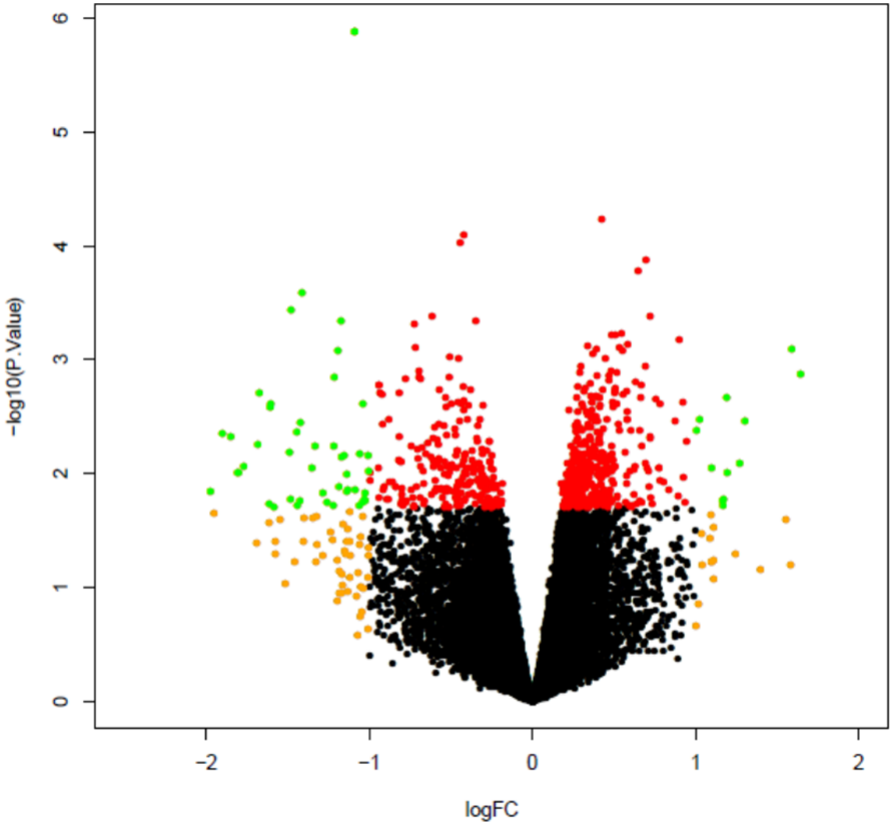
Volcano plot comparing plaque macrophage gene expression in infected mice as compared to mock infected mice. All microarray probes, both coding and non-coding, are represented. Volcano plot compares log2 (fold change) to log10 p value. Green dots represent differentially expressed genes as identified by FDG unadjusted p value < 0.05 and log2 fold change >1. Red dots represent probes with unadjusted p value < 0.05, log2 fold change ≤1 and orange dots p value ≥ 0.05, log2 fold change >1.

Next, plaque macrophages were collected at 2 weeks post infection (n=9) or mock infection (n=11) using LCM and RNA extracted from these cells to enable comparison of gene expression profiles between infected and mock infected mice. Analysis of microarray data using FDR adjusted p value identified only 1 differentially expressed probe which was non coding and therefore not assigned to a particular gene. The analysis was repeated using FDR unadjusted p value and identified 36 differentially expressed coding probes between infected and mock infected groups (**Figure 8**). These genes are listed in **Supplementary Table 3**.

In order to characterise the biological relevance of the 36 genes, pathway analysis was carried out using clusterProfiler to identify significantly perturbed KEGG pathways. Three pathways were significantly affected by pneumococcal infection: ubiquitin mediated proteolysis, endocytosis and HTLV-1 infection (**Supplementary Table 4**). All genes associated with these pathways were downregulated in plaque macrophages from the infected group. Both ubiquitin mediated proteolysis and endocytosis pathways were identified as significantly perturbed due to downregulation of 3 genes which coded for E3 ubiquitin ligases: anaphase promoting complex subunit 1 (Anapc1), HECT, UBA and WWE domain containing 1 (Huwe1) and Itch.

**Figure 9:**
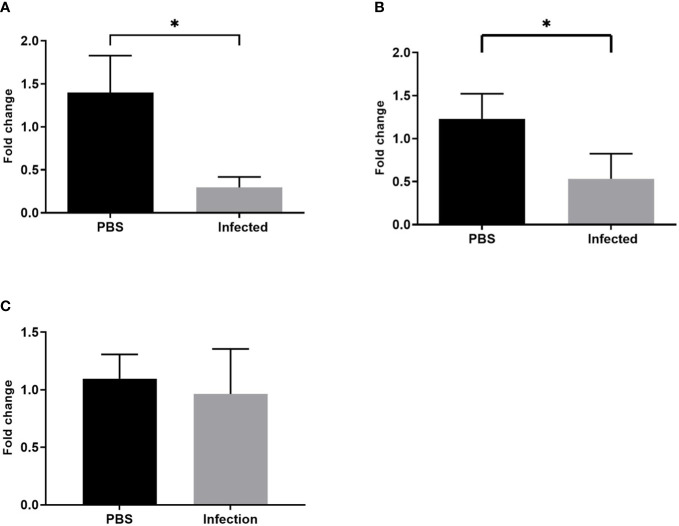
Pneumococcal infection leads to downregulation of Huwe1 and Itch expression in plaque macrophages. Gene expression (normalised to housekeeping gene 18s) of **(A)** Huwe1, **(B)** Itch and **(C)** Anapc1 in LCM derived plaque macrophages from infected mice compared with mock infected mice (*p<0.05, n=7).

To validate the microarray findings demonstrating downregulation of these E3 ubiquitin ligases, we performed qPCR analysis of LCM derived plaque macrophages from independent biological replicates 2 weeks post infection or mock infection. Down-regulation of Huwe1 and Itch was confirmed in infected mice using qPCR, but downregulation of Anapc1 was not demonstrated (**Figure 9**).

### 3.7 *De novo* statin use following pneumonia has no effect on markers of plaque vulnerability

**Figure 10:**
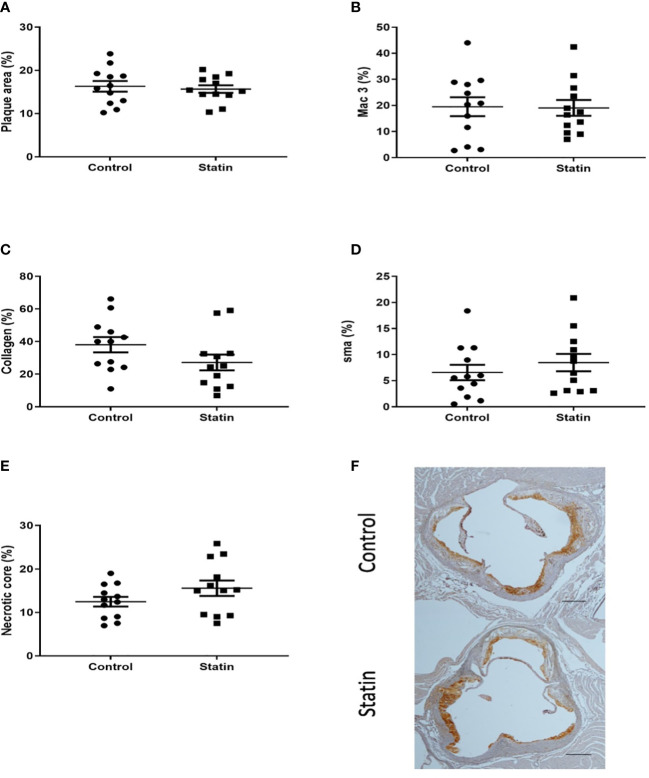
Atorvastatin had no effect on aortic sinus plaque size or composition 2 weeks post pneumonia. Quantification of aortic sinus plaque area **(A)**, plaque macrophage **(B)**, collagen **(C)**, smooth muscle **(D)** content and necrotic core area **(E)** (n=12). Representative images **(F)** of MAC-3 immunostained aortic sinus sections from a *S. pneumoniae* infected ApoE^-/-^ mouse (n=12) following atorvastatin therapy and an infected control mouse (n=12) given methylcellulose only. Scale bars represent 400 µm.

We next investigated whether the plaque stabilising capability of statins could be used to mitigate the previously demonstrated increase in plaque macrophage content following pneumonia. In the optimised model, mice were administered powdered atorvastatin calcium 40mg/kg in 0.5% methylcellulose or control (methylcellulose alone) once daily by gavage for 10 days, with the first statin dose administered 24 hours post infection. We observed no difference in aortic sinus plaque burden, plaque macrophage content or other markers of plaque vulnerability at 2 weeks post infection following statin therapy (**Figure 10**).

## 4 Discussion

Here, we developed a murine model of pneumococcal pneumonia on a background of established atherosclerotic plaque formation, with high rates of bacteremia and survival post infection, which can serve as a translational tool to further investigate the role of macrophages in post pneumonia plaque disruption and facilitate mechanistic studies. Infection resulted in an increase in plaque macrophage content 2 weeks post infection but no longer term effects on plaque composition were identified. Transcriptomic analysis of plaque macrophages suggested that very few genes were differentially expressed following infection, although our findings hint at a role for the ubiquitin-proteasome system (UPS) in the host response to pneumococcal infection within plaques. *De novo* initiation of atorvastatin following pneumonia had no effect on markers of plaque vulnerability to rupture.

Systemic inflammatory responses to pneumonia could contribute to plaque alterations which result in a greater vulnerability to rupture. Increased pneumonia severity, bacteremia and pre-existing coronary artery disease have been identified as risk factors for the development of MACE associated with pneumonia (Ramirez et al., 2008; Corrales-Medina et al., 2011; Corrales-Medina et al., 2012). We reasoned, therefore, that essential features for an optimized model to investigate changes in atherosclerotic plaques after pneumonia would include pneumonia, bacteremia, established atherosclerotic plaques at the time of pneumonia and survival for sufficient time to allow for observable changes in the plaque to occur. Extensive preliminary work was required to achieve the desired microbiological outcomes with high survival rates. Data generated during development of the model suggests that high fat diet fed ApoE^-/-^ mice may be more susceptible to severe pneumococcal infection compared to wild type mouse strains, which would be consistent with previous studies which have demonstrated that ApoE can protect against the development of sepsis (Rensen et al., 1997; de Bont et al., 2000; Laskowitz et al., 2000; Vonk et al., 2004). This is reflected in the fact that deaths were seen even in ApoE^-/-^ mice without bacteremia 24 hours post infection.

Using our new model, pneumococcal pneumonia resulted in increased plaque macrophage content in aortic sinus atherosclerotic plaques 2 weeks post infection, a feature which is associated with plaque vulnerability to rupture in humans (Virmani et al., 2006). However, pneumonia had no significant effect on the other measured markers of plaque vulnerability, which suggests that the prevailing phenotype of these plaque macrophages is such that they may have relatively little impact on the structural integrity of the plaque. It therefore remains open to question whether these macrophages have a detrimental effect on plaque stability, although it should be noted that plaques were examined at only two time points, and only after recovery from pneumonia. Histological examination of the atherosclerotic plaques in infected mice did not demonstrate significant cholesterol accumulation within the macrophages themselves and therefore their appearance was not consistent with them being foam cells.

There were no differences in plaque composition at the 8 week time point and, in contrast to data generated from pneumococcal infection in wild type mice (Denes et al., 2014), no differences in atherosclerotic lesion burden at either time point. Our results are in many ways consistent with findings from clinical observational studies of MI following pneumonia. Were pneumococcal pneumonia to result in an acceleration of atherosclerotic plaque formation, the risk of MI would be expected to continue to increase during this time (Calvert et al., 2011; Conte et al., 2017), whereas the clinical data generally demonstrate an initial peak in risk within the first few days post infection followed by a gradual decrease back towards baseline risk (Smeeth et al., 2004; Corrales-Medina et al., 2010; Musher et al., 2019). This suggests a reversal or mitigation of the mechanisms which can trigger MI post pneumonia, following the initial rapid increase in MI risk. If macrophages are involved in destabilising plaques post pneumonia, our finding of an increase in plaque macrophage content followed by a fall back to baseline levels would mirror the clinical data.

Clinical studies have suggested a long term effect on cardiovascular risk post pneumonia (O’Meara et al., 2005; Corrales-Medina et al., 2015), but in our model no significant differences in plaque composition were seen at the later 8 week time point. Our results suggest that a prolonged alteration in plaque vulnerability to rupture is unlikely to be the predominant mechanism that explains this persistent increase in cardiovascular risk. In keeping with this, despite evidence of increased inflammatory cell infiltration in ApoE^-/-^ mice atherosclerotic plaques following influenza infection, clinical studies suggest that increased cardiovascular risk following confirmed influenza infection is limited to only the first few days post infection (Kwong et al., 2018; Warren-Gash et al., 2018). Mechanisms which do not directly involve plaques which may explain the prolonged increase in cardiovascular risk include a persistent procoagulant state and prolonged impairment of myocardial function (Yende et al., 2011; Reyes et al., 2017). Pneumonia may therefore lead to persistently vulnerable blood and/or vulnerable myocardium, as opposed to vulnerable plaques (Naghavi et al., 2003). An important question in this field that remains unanswered is the proportion of post pneumonia MIs that are type 1 (coronary atherosclerotic plaque disruption) or type 2 (myocardial oxygen supply/demand mismatch) in nature. This answer to this question is critical to guide appropriate preventative and therapeutic strategies. If these are predominantly type 1 MIs, then the focus should be on plaque stabilisation, antiplatelet therapies and coronary revascularisation. Effective therapies for type 2 MIs have yet to be established, although it is likely that both prevention and management requires prompt correction of the process that has led to myocardial oxygen supply and demand mismatch (Thygesen and Jaffe, 2021). Although it has been proposed that most post pneumonia MIs term are predominantly type 1 in nature (Musher et al., 2019), this has yet to be confirmed in clinical studies. Impairment of myocardial function following pneumococcal pneumonia may increase the risk of both type 1 and type 2 MI (Brown et al., 2014; Reyes et al., 2017).

Identifying the exact mechanisms responsible for the initial increase in plaque macrophage content was beyond the scope of this study. Pneumococcal pneumonia had no effect on the proportion of plaque cells positive for Ki67, which suggests that cell proliferation did not significantly contribute to the accumulation of plaque macrophages, although dual immunostaining with a macrophage marker was not performed. The expansion of plaque macrophages at 2 weeks post infection was therefore likely due to increased recruitment of circulating monocytes and/or a reduction in plaque macrophage apoptosis. Infections caused by a variety of pathogens have been shown to induce Ly6C^hi^ monocyte mobilization from the bone marrow and recruitment to sites of infection (Shi and Pamer, 2011). In mouse atherosclerosis models, Ly6C^hi^ monocytes are predisposed to accumulating in plaques, play a key role in driving atherogenesis and are in general considered to be more inflammatory as compared to Ly6C^lo^ monocytes (Swirski et al., 2007; Soehnlein et al., 2013). Sepsis is associated with upregulation of endothelial cell adhesion molecules including ICAM-1 and VCAM-1, and this mechanism may also contribute to the accumulation of monocytes within plaques following pneumonia (Aird, 2003; Kaynar et al., 2014).

Transcriptomic analysis of plaque macrophages suggested that infection led to the differential expression of very few genes 2 weeks post pneumonia. In contrast, Guillon et al. found significant alterations in the transcriptome and phenotype of alveolar macrophages at least 4 weeks after pneumonia caused by serotype 19F *S. pneumoniae*, although these were C57BL/6 mice which underwent 2 rounds of infection 7 days apart (Guillon et al., 2020). However, they found that when only one lung was infected, remodelling of alveolar macrophages appeared to occur almost exclusively in the infected lung as compared to the contralateral lung, which suggests systemic signals during pneumococcal infection do not have a prolonged effect on the phenotype of macrophages distant to the site of infection.

Analysis of microarray data allowed us to identify 2 genes which were downregulated post infection. These genes encoded Huwe1 and Itch respectively, both of which are E3 ubiquitin ligases, part of the UPS. The UPS is an important degradation system in eukaryotic cells, and is involved in controlling the concentration of several regulatory proteins as well as terminating damaged proteins (Tanaka and Matsuda, 2014). Our data hints at a role for the UPS in the inflammatory response to pneumonia in the plaque microenvironment. Analysis of plaque macrophages at an earlier post infection time point may reveal greater perturbation in UPS related pathways.

Macrophage apoptosis plays a significant role in the host response to pneumococcal infection by contributing to late phase bacterial killing (Preston et al., 2019). Following pneumococcal infection, Huwe1 promotes macrophage apoptosis by ubiquitinating the anti-apoptotic protein Mcl-1 and therefore targeting it for degradation by the proteasome (Bewley et al., 2011). Downregulation of Huwe1 by atherosclerotic plaque macrophages may therefore inhibit macrophage apoptosis and could explain the expansion of macrophages seen in the post pneumonic plaque. Small interfering RNA silencing of Huwe1 in THP-1 macrophages has been shown to increase ABCG1 mediated cholesterol efflux, although we found no evidence of increased cholesterol efflux in our model as both necrotic core size and plaque burden were unaffected by pneumonia (Aleidi et al., 2015). Genetic deficiency of Itch in ApoE^-/-^ mice has been shown to reduce atherosclerotic plaque formation primarily by preventing the degradation of Sterol regulatory element-binding protein 2 and thus upregulating LDL receptor expression leading to increased LDL reuptake in the liver (Stohr et al., 2015). Itch has been shown to play a role in the regulation of T cell activation, but determining any effect of its downregulation by macrophages on their interaction with plaque T cells post pneumonia requires further work (Poels et al., 2020). Evidence from human studies has demonstrated accumulation of ubiquitin conjugates in unstable coronary and carotid plaques, suggesting that increased ubiquitination may itself be a marker of plaque vulnerability (Herrmann et al., 2002; Versari et al., 2006). Downregulation of both Huwe1 and Itch would overall be expected to lead to a reduction in plaque burden and possibly plaque vulnerability. The fact we did not find evidence for this effect in our model may be due to the downregulation not being of sufficient magnitude or not being sustained for a sufficient period of time, or that the downregulation may be compensatory following a period of increased expression earlier in the post infection period. Proteomic evaluation is required to confirm an alteration in the levels of these E3 ubiquitin ligases following infection.

Statins have been shown to have plaque stabilising effects in both animal and clinical studies (Crisby et al., 2001; Hattori et al., 2012; Nie et al., 2014). These effects have mostly been found after several months of statin therapy, although Larmann et al. demonstrated a reduction in plaque macrophage content after only 4 days of atorvastatin therapy in ApoE^-/-^ mice which had been fed an atherogenic diet for 16 weeks (Larmann et al., 2013). Using our model we found that *de novo* initiation of atorvastatin following pneumonia had no impact on plaque macrophage content or any other markers of vulnerability. The atorvastatin dose used (40 mg/kg/d) is equivalent to 227 mg/d in a 70kg human based on body surface area, which exceeds the highest recommended daily human dose (80mg) (Reagan-Shaw et al., 2008). If increased risk of plaque rupture plays an integral role in early post pneumonia MI, our data suggest that statins commenced soon after onset of pneumonia are unlikely to reduce this risk. However, they may be able to reduce short term cardiovascular risk following infection due to pleiotropic anticoagulant and antiplatelet effects or through beneficial effects on endothelial function (Landmesser et al., 2005; Violi et al., 2013). Statins may also improve neutrophil function during the immune response to pneumonia (Sapey et al., 2019).

A clear limitation of the ApoE^-/-^ mouse model is that both spontaneous plaque rupture and plaque development within the coronary artery rarely occur and therefore these mice do not develop plaque associated MI (Oppi et al., 2019). In recent years the Apolipoprotein E-deficient Fibrillin-1 mutant (ApoE-/-Fbn1C1039G+/-) mouse strain has been shown to develop advanced plaques which can spontaneously rupture and result in MI (Van der Donckt et al., 2015). These mice may prove to be useful in the investigation of plaque rupture events following pneumonia in future studies. The relatively young age of the mice used in our model is another important caveat to our findings. Aged mice demonstrate impaired bacterial clearance and an altered immune response to pneumococcal infection as compared to younger mice, including increased levels of serum tumour necrosis factor, greater expansion of circulating Ly6C^hi^ monocytes and increased tissue recruitment of these monocytes (Puchta et al., 2016). Ageing in humans has been associated with innate immune system dysfunction (Shaw et al., 2013) and older age has been identified as a risk factor for post-pneumonia MI in clinical studies (Viasus et al., 2013). Other limitations include not assessing the effect of pneumonia on other immune cells that may be found in plaques (including T lymphocytes and neutrophils), not assessing the systemic cytokine response to pneumonia in our model, investigating the effect of only one *S. pneumoniae* serotype and only using male mice in the final model. In contrast to our findings, abdominal sepsis in ApoE^-/-^ mice aged 22-24 weeks which had been fed an atherogenic diet for 16 weeks prior to infection resulted in an acceleration of atherosclerotic plaque formation over a 5 month period (Kaynar et al., 2014). This abdominal sepsis model further differed from ours in that atherogenic diet was continued following infection.

In conclusion, we have developed a novel murine model of pneumococcal pneumonia on a background of established atherosclerosis. Using this model, we found evidence of increased plaque macrophage content at two 2 weeks post infection, which may increase plaque vulnerability to rupture, although we found no impact on other plaque vulnerability markers at this time point. Our results suggest that pneumonia had no longer term effect on plaque composition or burden. Transcriptomic analysis of plaque macrophages demonstrated downregulation of two E3 ubiquitin ligases which hints at a role for the UPS system in the response to pneumococcal infection in the plaque microenvironment. Our model is amenable to simple adjustments with regard to severity of pneumonia and atherosclerotic burden at the time of infection. It can be used as a foundation to further explore the effect of pneumococcal pneumonia, the most common cause of CAP worldwide, on atherosclerotic plaque composition and further our understanding of the link between pneumonia and MI through future mechanistic investigation. This can provide a valuable adjunct with which to inform translational studies in man that target interventions after pneumonia to reduce the burden of subsequent cardiovascular disease.

## Data availability statement

The datasets presented in this study can be found in online repositories. The names of the repository/repositories and accession number(s) can be found below: https://www.ebi.ac.uk/arrayexpress/E-MTAB-11462.

## Ethics statement

The animal studies were reviewed and approved by the animal project review committee, University of Sheffield.

## Author contributions

RB, HM, SF and DD designed the experiments. RB, HM, CW, JC, LW, CG and PH were involved in data collection and curation. RB, PH, AM-D, SF and DD were involved in data analysis. RB, SF and DD wrote the original draft. All authors contributed to the article and approved the submitted version.
