# Supplementary material for: Transient increase in atherosclerotic plaque macrophage content following Streptococcus pneumoniae pneumonia in ApoE-deficient mice
Source: Front Cell Infect Microbiol. 2023 Mar 23;13:1090550. doi: 10.3389/fcimb.2023.1090550 (PMC10076735; doi:10.3389/fcimb.2023.1090550)
Supplement: Supplementary file 1 [file DataSheet_1.docx]

**Transient increase in atherosclerotic plaque macrophage content following *Streptococcus pneumoniae* pneumonia – Supplementary data**

**Supplementary figures**

**Supplementary Figure 1**. **Blood viable bacterial counts in male, Western diet fed ApoE^-/-^ mice 24 hours after intranasal instillation of serotype 4 *S. pneumoniae*.** Bacteria in blood following infection with 10^5^, 5 x 10^4^ or 10^4^ cfu serotype 4 pneumococci demonstrating consistent bacteremia following the 10^5^ cfu dose (n=3-5).


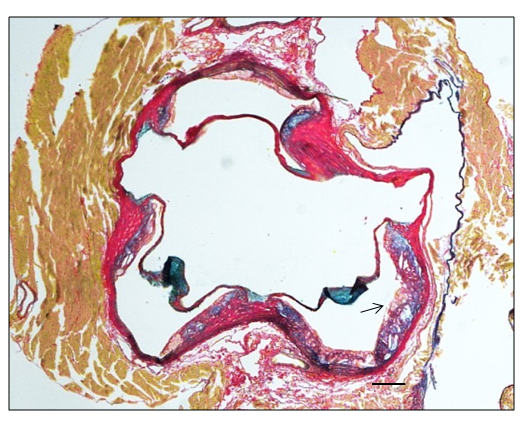


**Supplementary Figure 2. Aortic sinus following 8 weeks of Western diet feeding.** Representative image (n=5) of Miller’s Elastin/Modified Van Gieson stained aortic sinus section from a male ApoE^-/-^ mouse fed Western diet for 8 wks. Arrow points to advanced atheromatous lesion (scale bar 200 µm).


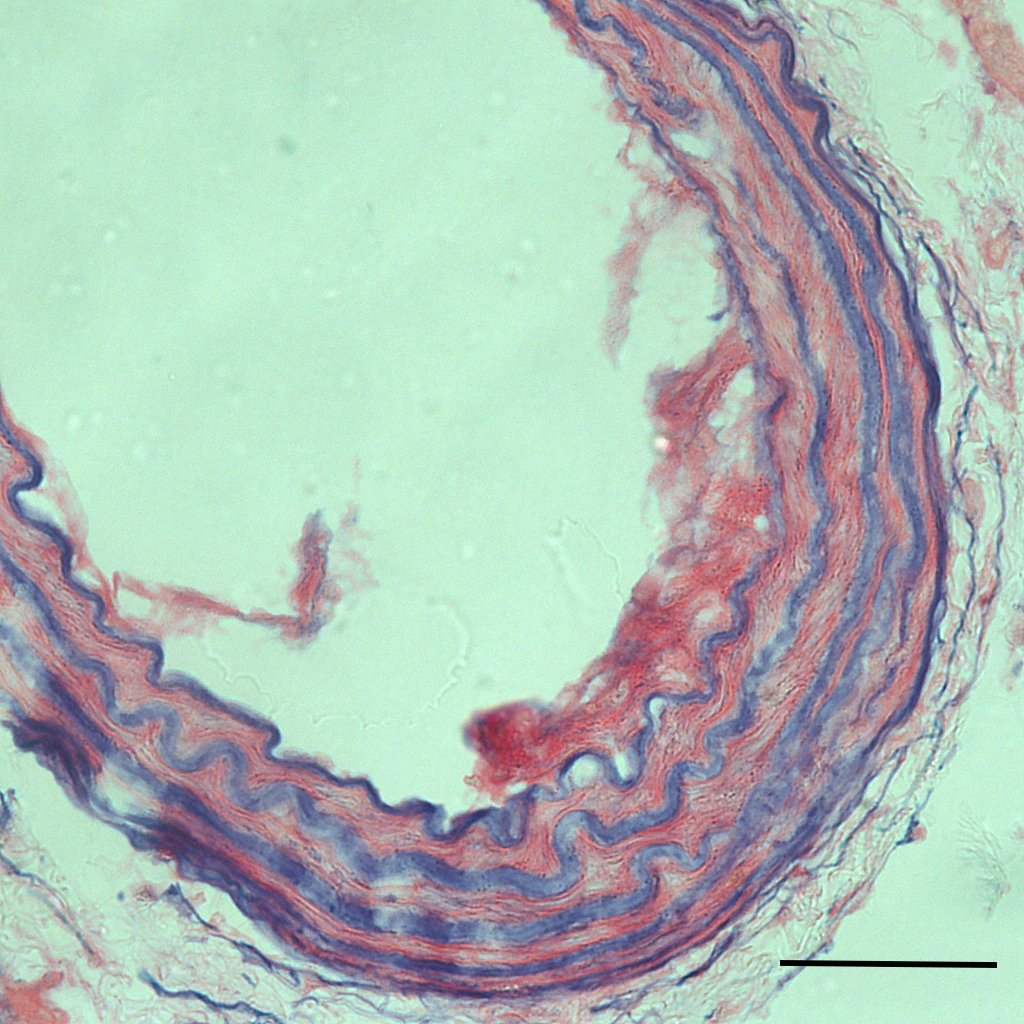


**Supplementary Figure 3. Brachiocephalic artery section 2 weeks post infection.** Representative image (n=10) of Miller’s Elastin/Modified Van Gieson stained brachiocephalic artery section from a male ApoE^-/-^ mouse fed Western diet for 8 wks followed by intranasal instillation of 5x10^5^ cfu serotype 4 (TIGR4) *S. pneumoniae* and subsequent antibiotic administration consisting of three doses of s.c. 100 mg/kg amoxicillin 12 hourly commenced 24 h after infection (scale bar 100 µm).


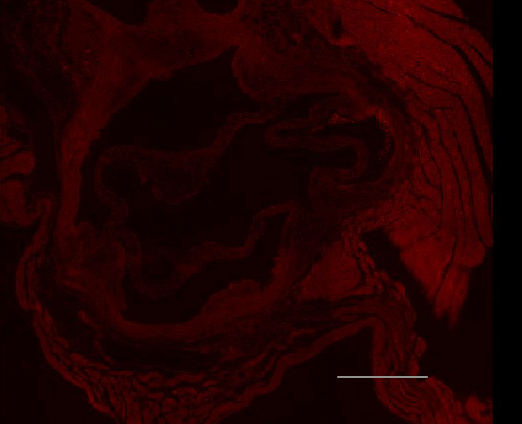


**Supplementary Figure 4. Aortic sinus section 2 weeks post infection.** Immunofluorescence assay using anti-serotype 4 pneumococcal antiserum showed no positive staining in atherosclerotic plaques or elsewhere in the aortic sinus 2 weeks post pneumococcal infection using our optimised model (n=6) (scale bar 400 µm).

Supplementary Figure 5. Enrichment of lesion macrophage RNA following LCM

qPCR analysis comparing expression of macrophage-specific marker CD68 (A) and smooth muscle marker ACTA2 (B) in LCM isolated plaque macrophage RNA compared with RNA extracted from whole aortic sinus sections (normalised to cyclophilin A), n=4.

**Supplementary methods**

**Immunohistochemistry**

Aortic sinus sections were dewaxed in xylene, before rehydration through graded alcohols to water. Endogenous peroxidases were blocked with hydrogen peroxide (30% v/v; Sigma-Aldrich, UK) and sections were then incubated in a solution of 0.1% w/v milk buffer (commercially available Marvel skimmed milk powder) in PBS for 30 minutes to block non-specific binding of the secondary antibody. An antigen retrieval step was performed if necessary, after which sections were incubated with primary antibody overnight at 4°C or for 1 hour at room temperature. Binding of the primary antibody to the target antigen was identified indirectly by incubating sections with biotinylated secondary antibody (1:200 dilution in PBS) for 30 minutes at room temperature, followed by avidin-biotinylated enzyme complexes with horseradish peroxidase (HRP) (Vectastain ABC-HRP kit , Vector Labs, UK) and finally the HRP substrate SigmaFAS 3,3′- diaminobenzidine tetrahydrochloride (DAB) (Sigma-Aldrich). Sections were counterstained with Carazzi’s haematoxylin, dehydrated through graded alcohols into xylene and finally mounted under coverslips with DPX. Antibodies used for immunohistochemistry are summarised in Supplementary Table 1.

| **Primary antibody** | **Secondary antibody** | **Antigen retrieval** | **Dilution** | **Incubation** |
| --- | --- | --- | --- | --- |
| MAC-3 (rat anti- mouse; BD Biosciences 553322) | Biotinylated anti-rat IgG (Vector Laboratories BA-9400) | Citrate buffer | 1:500 | Overnight at 4°C |
| α-smooth muscle actin (mouse monoclonal anti-human M0851, Dako) | Biotinylated anti-mouse IgG (Vector Laboratories BA-9200) | None | 1:150 | 1 hour at RT |
| Ki67 (rabbit monoclonal, Abcam, ab1667) | Biotinylated anti-rabbit IgG (Vector Laboratories BA-1000) | Citrate buffer | 1:200 | Overnight at 4°C |

**Supplementary** **Table 1.** Antibodies used for immunohistochemistry.

### MAC-3 immunostaining of cryo-sections

Immunohistochemical staining for the macrophage marker MAC-3 was performed on every 5^th^ aortic sinus cryo-section to be used as guide slides to direct LCM of plaque macrophages on the remaining Toluidine blue stained cryo-sections. Sections were fixed in ice cold acetone for 10 minutes, then rinsed in 3 changes of PBS followed by incubation for 10 minutes with Bloxall (Vector Laboratories, UK), an endogenous peroxidase and alkaline phosphatase blocking solution, before rinsing again in 3 changes of PBS. Slides were incubated in 4% v/v rabbit serum (Vector Laboratories) in PBS for 15 minutes then incubated in primary antibody (rat anti-mouse MAC-3 (BD Biosciences 550292) 1:200 dilution in 4% rabbit serum) for 1 hour at room temperature, then rinsed in 3 changes of PBS. Sections were incubated in secondary antibody (biotinylated rabbit anti-rat IgG mouse adsorbed antibody (Vector Laboratories, BA-4001) 1:200 dilution in 4% rabbit serum) for 15 minutes, followed by Vectastain ABC-HRP and then DAB before counterstaining with Carazzi’s haematoxylin and dehydration through graded alcohols into xylene and finally mounted under coverslips with DPX.

### Type 4 pneumococcal antiserum immunofluorescence staining protocol

Antibodies were diluted in 10% v/v normal goat serum (Vector Laboratories, UK) in PBS. Slides were dewaxed in xylene for 10 minutes then rehydrated through graded alcohols (100%, 90%, 70% and 50% v/v ethanol for 2 minutes each step) through to water. Slides underwent citrate antigen retrieval by being incubated in citrate buffer for 20 minutes at 95°C, then cooled for 20 minutes at room temperature before being rinsed in PBS. Slides were then incubated with 1% w/v skimmed milk powder in PBS for 30 minutes. Excess milk buffer was carefully blotted away from around tissue sections. Slides were then incubated with primary rabbit anti-serotype 4 pneumococcus antiserum 16747 (1:000 dilution; Statens Serum Institute, Denmark) for 1 hour at room temperature, before being rinsed in 3 changes of PBS, 5 minutes each. Sections were then incubated with fluorescent secondary antibody Alexa Fluor 568 goat anti-rabbit IgG (1:1500 dilution, Invitrogen, UK) in the dark for 1 hour at room temperature. Slides were then rinsed in 3 changes of PBS, 5 minutes each. Sections were mounted with coverslips using Vectashield hard set mounting medium with DAPI (Vector Laboratories, UK) and stored in the dark.

| **Gene** | **Primer (Life Technologies, UK)** |
| --- | --- |
| 18S | Mm03928990_g1 |
| Ppia (Cyclophilin A) | Mm02342430_g1 |
| CD68 | Mm03047343_m1 |
| Acta 2 (smooth muscle actin) | Mm01204962_gH |
| Itch | Mm00492683_m1 |
| Anapc1 | Mm01336123_m1 |
| Huwe1 | Mm00615533_m1 |

**Supplementary Table 2.** mRNA qPCR assay primers

| **Affymetrix probe**  **ID** | **Gene Symbol** | **Log2 Fold change** | **p value** | **Gene name** |
| --- | --- | --- | --- | --- |
| 17484100 | zinc finger, RAN-binding domain containing 1 | -1.85063 | 0.004772 | Zranb1 |
| 17391480 | anaphase promoting complex subunit 1 | -1.8085 | 0.009954 | Anapc1 |
| 17538773 | HECT, UBA and WWE domain containing 1 | -1.77086 | 0.008573 | Huwe1 |
| 17359961 | golgi-specific brefeldin A-resistance factor 1 | -1.68328 | 0.005597 | Gbf1 |
| 17221250 | ADP-ribosylation factor guanine nucleotide-exchange factor 1(brefeldin A-inhibited) | 1.642493 | 0.001327 | Arfgef1 |
| 17300371 | predicted gene 20521 | -1.61363 | 0.018606 | Gm20521 |
| 17300185 | olfactory receptor 204 | -1.58533 | 0.019681 | Olfr204 |
| 17221233 | ADP-ribosylation factor guanine nucleotide-exchange factor 1(brefeldin A-inhibited) | 1.554567 | 0.025784 | Arfgef1 |
| 17378318 | itchy, E3 ubiquitin protein ligase | -1.48962 | 0.006535 | Itch |
| 17206483 | small nucleolar RNA, H/ACA box 28 | -1.48322 | 0.016768 | Snora28 |
| 17359926 | golgi-specific brefeldin A-resistance factor 1 | -1.47904 | 0.000368 | Gbf1 |
| 17324680 | discs, large homolog 1 (Drosophila) | -1.44446 | 0.00436 | Dlg1 |
| 17202517 | olfactory receptor 1313 | -1.42432 | 0.017611 | Olfr1313 |
| 17206727 | ankyrin repeat domain 17 | -1.35285 | 0.008902 | Ankrd17 |
| 17290927 | zinc finger with KRAB and SCAN domains 3 | -1.33248 | 0.005763 | Zkscan3 |
| 17359948 | golgi-specific brefeldin A-resistance factor 1 | -1.32392 | 0.02368 | Gbf1 |
| 17308472 | exportin 7 | 1.301569 | 0.003501 | Xpo7 |
| 17219478 | coatomer protein complex subunit alpha | -1.28481 | 0.014817 | Copa |
| 17200103 | coatomer protein complex, subunit gamma 1 | 1.269288 | 0.008141 | Copg1 |
| 17449547 | ankyrin repeat domain 17 | -1.23804 | 0.032238 | Ankrd17 |
| 17403846 | vomeronasal 1 receptor 12 | -1.22049 | 0.018813 | Vmn1r12 |
| 17458948 | olfactory receptor 1504 | -1.21671 | 0.005725 | Olfr1504 |
| 17202979 | olfactory receptor 1135 | 1.194902 | 0.009684 | Olfr1135 |
| 17221253 | ADP-ribosylation factor guanine nucleotide-exchange factor 1(brefeldin A-inhibited) | -1.18669 | 0.013091 | Arfgef1 |
| 17341696 | 3-phosphoinositide dependent protein kinase 1 | -1.17206 | 0.000454 | Pdpk1 |
| 17348452 | RIO kinase 3 | -1.1686 | 0.007253 | Riok3 |
| 17484102 | zinc finger, RAN-binding domain containing 1 | -1.16181 | 0.028292 | Zranb1 |
| 17202919 | calnexin | -1.1541 | 0.006838 | Canx |
| 17220754 | angel homolog 2 (Drosophila) | -1.13319 | 0.030408 | Angel2 |
| 17391446 | anaphase promoting complex subunit 1 | -1.13013 | 0.013971 | Anapc1 |
| 17308463 | exportin 7 | -1.11946 | 0.040084 | Xpo7 |
| 17207455 | apolipoprotein L 7c | 1.111031 | 0.030042 | Apol7c |
| 17219451 | coatomer protein complex subunit alpha | -1.05955 | 0.042363 | Copa |
| 17359958 | golgi-specific brefeldin A-resistance factor 1 | -1.02617 | 0.014976 | Gbf1 |
| 17341704 | 3-phosphoinositide dependent protein kinase 1 | 1.02562 | 0.003371 | Pdpk1 |
| 17238667 | C-type lectin domain family 4, member e | -1.00679 | 0.006966 | Clec4e |

Supplementary Table 3. Differentially expressed genes (unpaired t test <0.05) in plaque macrophages in *S. pneumoniae* infected mice as compared to mock infected mice.

| KEGG category | KEGG subcategory | KEGG pathway | Adjusted p value | Genes |
| --- | --- | --- | --- | --- |
| Genetic Information processing | Folding, sorting and degradation | Ubiquitin mediated proteolysis | 0.011337 | Anapc1, Huwe1, Itch |
| Cellular processes | Transport and catabolism | Endocytosis | 0.030526 | Anapc1, Huwe1, Itch |
| Human diseases | Infectious diseases:viral | HTLV-I infection | 0.030526 | Canx, Dlg1, Anapc1 |

**Supplementary Table 4.** KEGG pathways, and their categories, altered by pneumococcal infection in plaque macrophages.
